# Publisher Correction: Long-term culture of human pancreatic slices as a model to study real-time islet regeneration

**DOI:** 10.1038/s41467-020-17574-x

**Published:** 2020-07-22

**Authors:** Mirza Muhammad Fahd Qadir, Silvia Álvarez-Cubela, Jonathan Weitz, Julia K. Panzer, Dagmar Klein, Yaisa Moreno-Hernández, Sirlene Cechin, Alejandro Tamayo, Joana Almaça, Helmut Hiller, Maria Beery, Irina Kusmartseva, Mark Atkinson, Stephan Speier, Camillo Ricordi, Alberto Pugliese, Alejandro Caicedo, Christopher A. Fraker, Ricardo Luis Pastori, Juan Domínguez-Bendala

**Affiliations:** 1 Diabetes Research Institute, University of Miami Miller School of Medicine, Miami, FL 33136 USA; 2 Department of Cell Biology and Anatomy, University of Miami Miller School of Medicine, Miami, FL 33136 USA; 3 Department of Medicine, Division of Metabolism, Endocrinology and Diabetes, University of Miami Miller School of Medicine, Miami, FL 33136 USA; 4 Universidad Francisco de Vitoria, Madrid, Spain; 5 nPOD Laboratory, Department of Pathology, Immunology and Laboratory Medicine, University of Florida, Gainesville, FL 32611 USA; 6 Department of Pathology, Immunology and Laboratory Medicine, College of Medicine, University of Florida, Gainesville, FL 32611 USA; 7 Paul Langerhans Institute Dresden (PLID) of the Helmholtz Zentrum München at the University Clinic Carl Gustav Carus of Technische Universität Dresden, Helmholtz Zentrum München, Neuherberg, Germany; 8 Faculty of Medicine, Institute of Physiology, Technische Universität Dresden, Dresden, Germany; 9 German Center for Diabetes Research (DZD), München, Neuherberg Germany; 10 Department of Surgery, University of Miami Miller School of Medicine, Miami, FL 33136 USA; 11 Department of Microbiology & Immunology, University of Miami Miller School of Medicine, Miami, FL 33136 USA; 12 Department of Biomedical Engineering, University of Miami Miller School of Medicine, Miami, FL 33136 USA

**Keywords:** Biological models, Tissue culture, Regenerative medicine, Stem-cell niche, Regeneration

Correction to: *Nature Communications* 10.1038/s41467-020-17040-8, published online 29 June 2020.

The original version of this Article was updated shortly after publication, to replace an incorrect version of the Peer Review File. The error has now been fixed and the Peer Review File PDF is available to download from the HTML version of the Article.

