# Supplementary material for: Long-term culture of human pancreatic slices as a model to study real-time islet regeneration
Source: Nat Commun. 2020 Jun 29;11:3265. doi: 10.1038/s41467-020-17040-8 (PMC7324563; doi:10.1038/s41467-020-17040-8)
Supplement: Supplementary file 1 — Supplementary Information [file 41467_2020_17040_MOESM1_ESM.pdf]

SUPPLEMENTAL INFORMATION

Qadir et al, 2020

1. Supplementary Figures 1-5
2. Supplementary Tables 1-2

Supplementary Figure 1

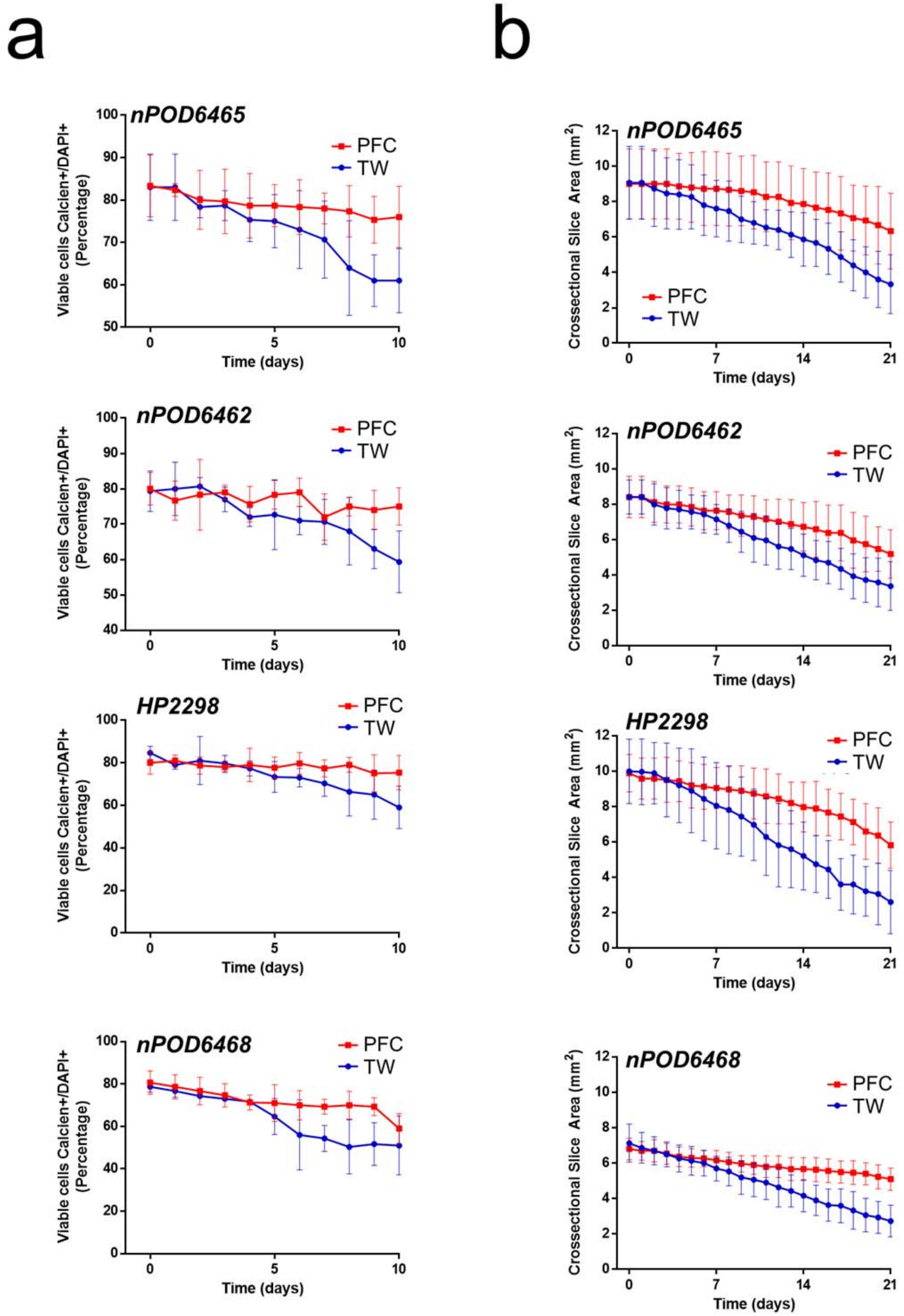

Supplementary Figure 1. Human pancreatic slices remain viable after extended culture atop PFC dishes.

(a) Viable cell percentage of human pancreatic slices cultured atop PFC-based and transwell membranes as a function of time (10 days). Red: PFC; blue: transwells (*n* = 4). **(b)** Cross-sectional area (sq. mm) of HPSs over

21 days of culture atop PFC/Si and transwell dishes. Red: PFC; blue: transwells. De-identified sample numbers from nPOD or the Diabetes research Institute's cGMP facility are indicated for each graphic (n = 4). Data in (**a-b**) are presented as mean ± SD. Each n further represents the mean of 3 technical replicates, while plotted bars/lines are centered at mean.

Supplementary Figure 2

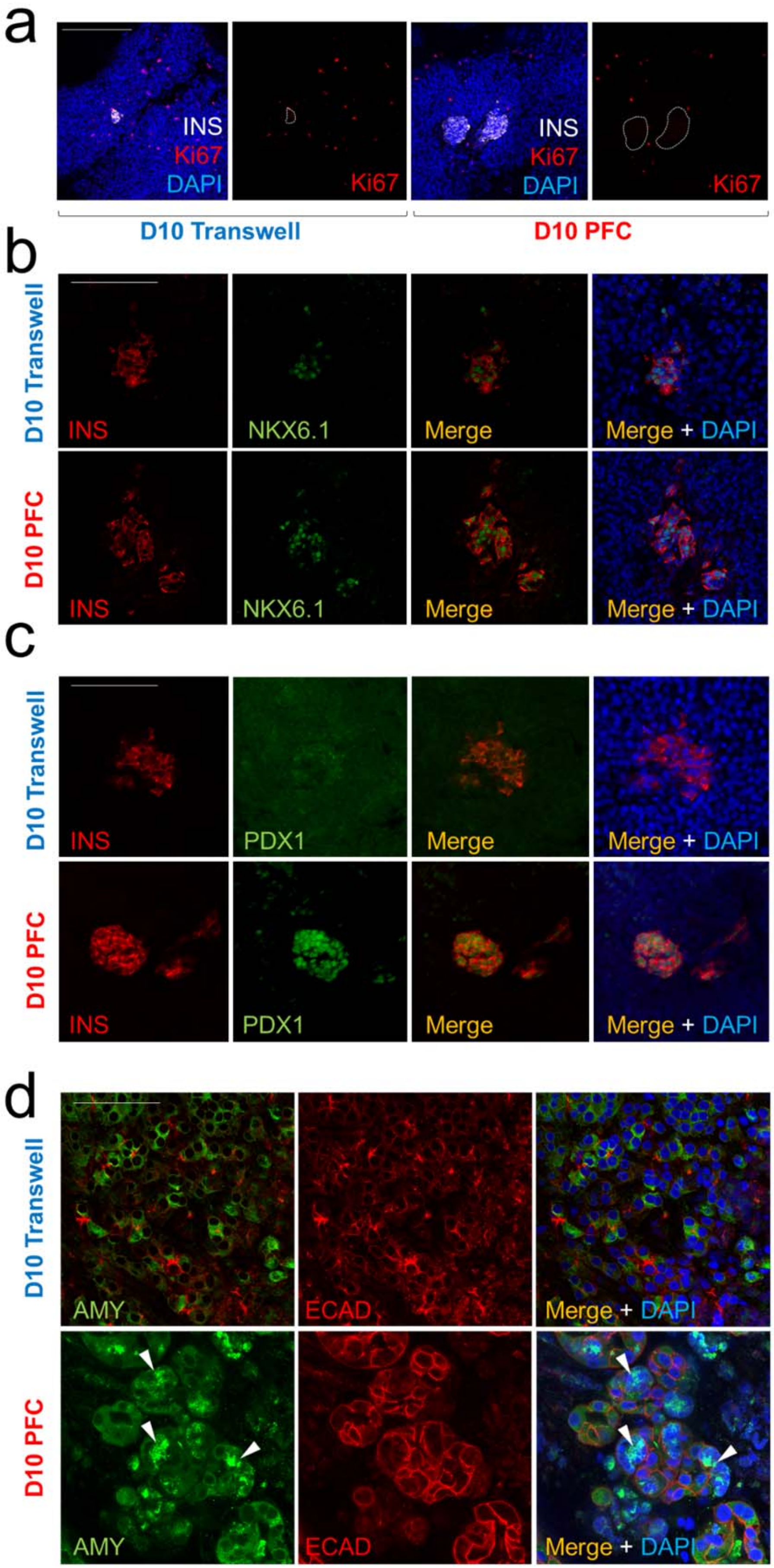

Supplementary Figure 2. Immunofluorescence (IF) analysis of long-term cultured slices shows differences in key epithelial, endocrine and acinar markers between transwell and PFC conditions, but not in β-cell turnover. **(a)** Representative Ki67 (red) IF microphotograph of transwell- (first and second panels) and PFC-cultured (second and third panels) human pancreatic slices. Insulin (INS) is shown in light grey, and nuclear counterstaining (DAPI), in blue. Most $Ki67^+$ cells were found in the exocrine compartment, with no apparent differences between either condition (*n* = 3). **(b)** Representative IF microphotograph of NKX6.1 (green) in islets (INS, red) of transwell- (top row) and PFC-cultured (bottom row) human pancreatic slices. Nuclear counterstaining: DAPI (blue) (*n* = 3). **(c)** Representative IF microphotograph of PDX1 (green) in islets (INS, red) of transwell- (top row) and PFC-cultured (bottom row) human pancreatic slices. Nuclear counterstaining: DAPI (blue). PDX1 is barely detectable in transwell-cultured slices after 10 days (*n* = 3). **(d)** Representative IF microphotograph of amylase (AMY, green) and E-cadherin (ECAD, red) in human pancreatic slices cultured in transwells (top row) and PFC (bottom row). Nuclear counterstaining: DAPI (blue). Slices cultured in PFC exhibit a better-preserved E-cadherin acinar tissue ultrastructure and more defined apical granularity of amylase granules (white arrows) than those cultured in transwells (*n* = 3). Size bars: 200 μm **(a)**; 100 μm **(b, c)**; and 50 μm **(d)**. Each *n* further represents the mean of 3 technical replicates.

Supplementary Figure 3

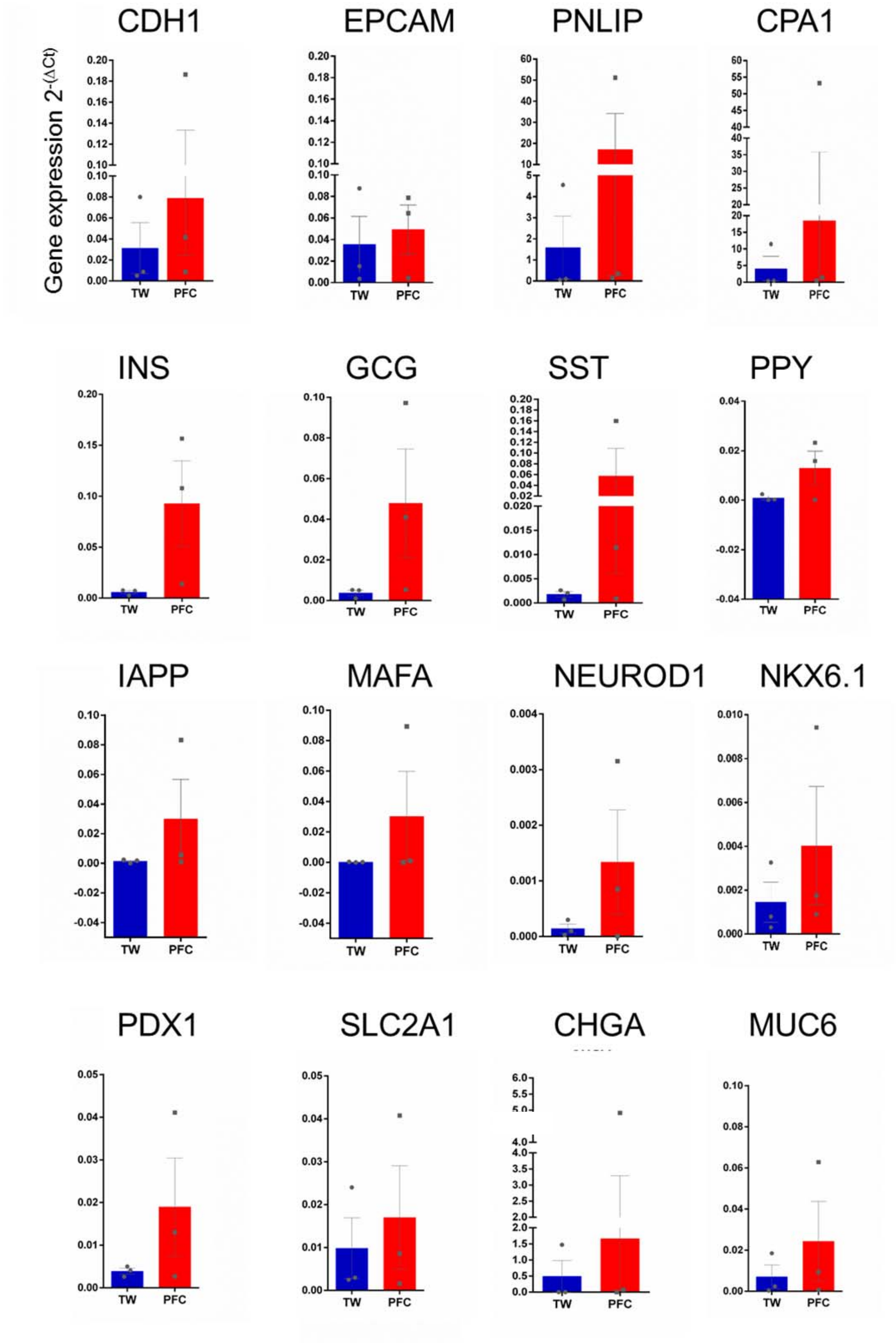

Supplementary Figure 3. qRT-PCR of key pancreatic endocrine and non-endocrine genes in transwell- or PFC-cultured human pancreatic slices at day 10. Values are represented as $2^{-(\Delta Ct)}$ (*y*-axis) for both transwell-

(blue columns) and PFC-cultured (red columns) slices. Each data point is the average of 3 technical replicates for one individual donor (n=3 biologically independent samples from individual donors). In order to compare the PFC and TW for each gene, we used a two-tailed Wilcoxon rank test. The p-value significance threshold was defined as 0.05. Owing to high donor-to-donor variability, and despite consistently higher values in PFC than in transwell conditions, the differences were not statistically significant by two-tailed *t*-test. Individual *p*-values: CDH1: p=0.4880; EPCAM: p=0.7107; PNLIP: p=0.4541; CPA1: p=0.4997; INS p=0.1734; GCG: p=0.2401; SST: p=0.3917; PPY p=0.2158; IAPP: p=0.3958; MAFA: p=0.4174; NEUROD1: p=0.3309; NKX6.1: p=0.3194; PDX1: p=0.4671; SLC2A2: p=0.6420; CHGA: p=0.5517; MUC6: p=0.4730. Normalizer housekeeping gene: B2M. Data plotted are presented as mean ± SD. Each n further represents the mean of 3 technical replicates, while plotted bars are centered at mean.

Supplementary Figure 4

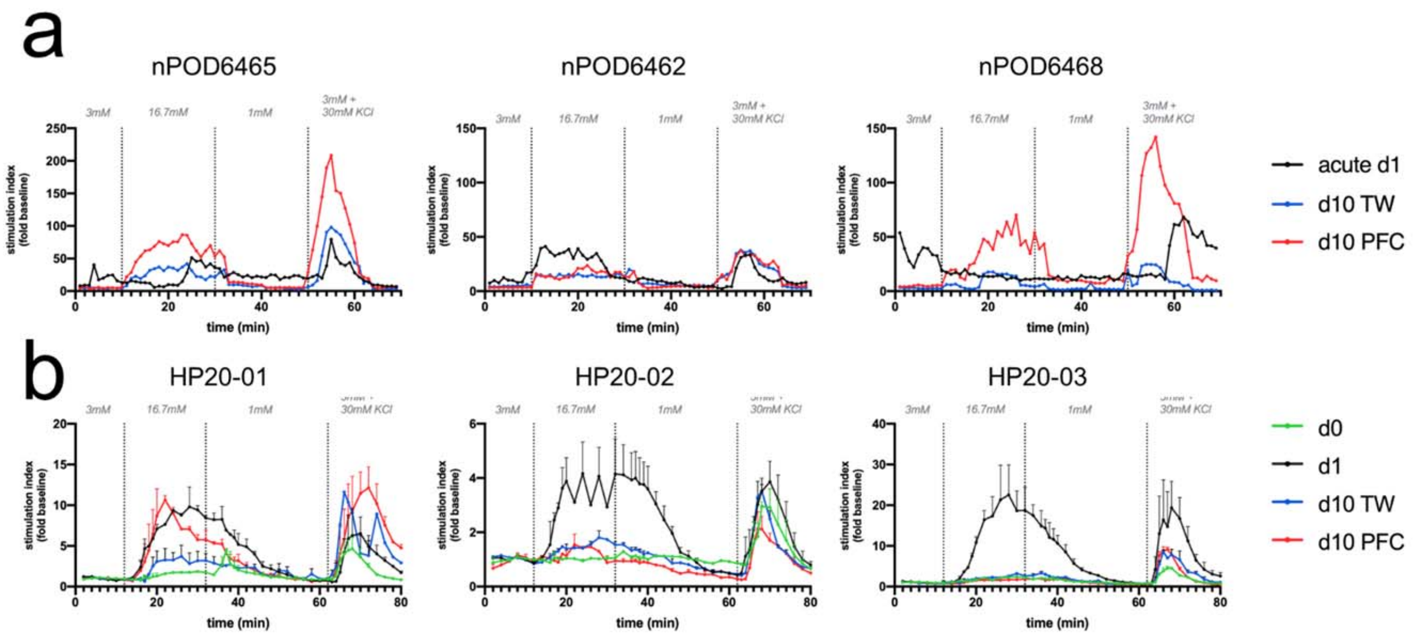

Supplementary Figure 4. Dynamic insulin secretion from human pancreatic slices. (a) Donor-by-donor profiles of insulin secretion [presented as stimulation index (SI) vs. baseline] after stimulation with 16.7 mM Glucose and 30 mM KCl. Preparations (*n*=3) used for **Fig. 3a**. For all graphics in this figure, black traces correspond to day +1^{S} (slices shipped from Gainesville); red: PFC-cultured slices for 10 days; and blue: transwell-cultured slices for 10 days. **(b)** Preparations (*n*=3) used for **Fig. 3g**. For all graphics in this figure, green is day 0 (perifusion immediately after sectioning); black is day +1 (perifusion after 24h rest); red: PFC-cultured slices for 10 days; and blue: transwell-cultured slices for 10 days. De-identified sample numbers are indicated for each graphic. Data plotted in (a-b) are represented by mean ± S.E.M. Each *n* further represents the mean of 3 technical replicates, while plotted lines are centered at mean.

Supplementary Figure 5

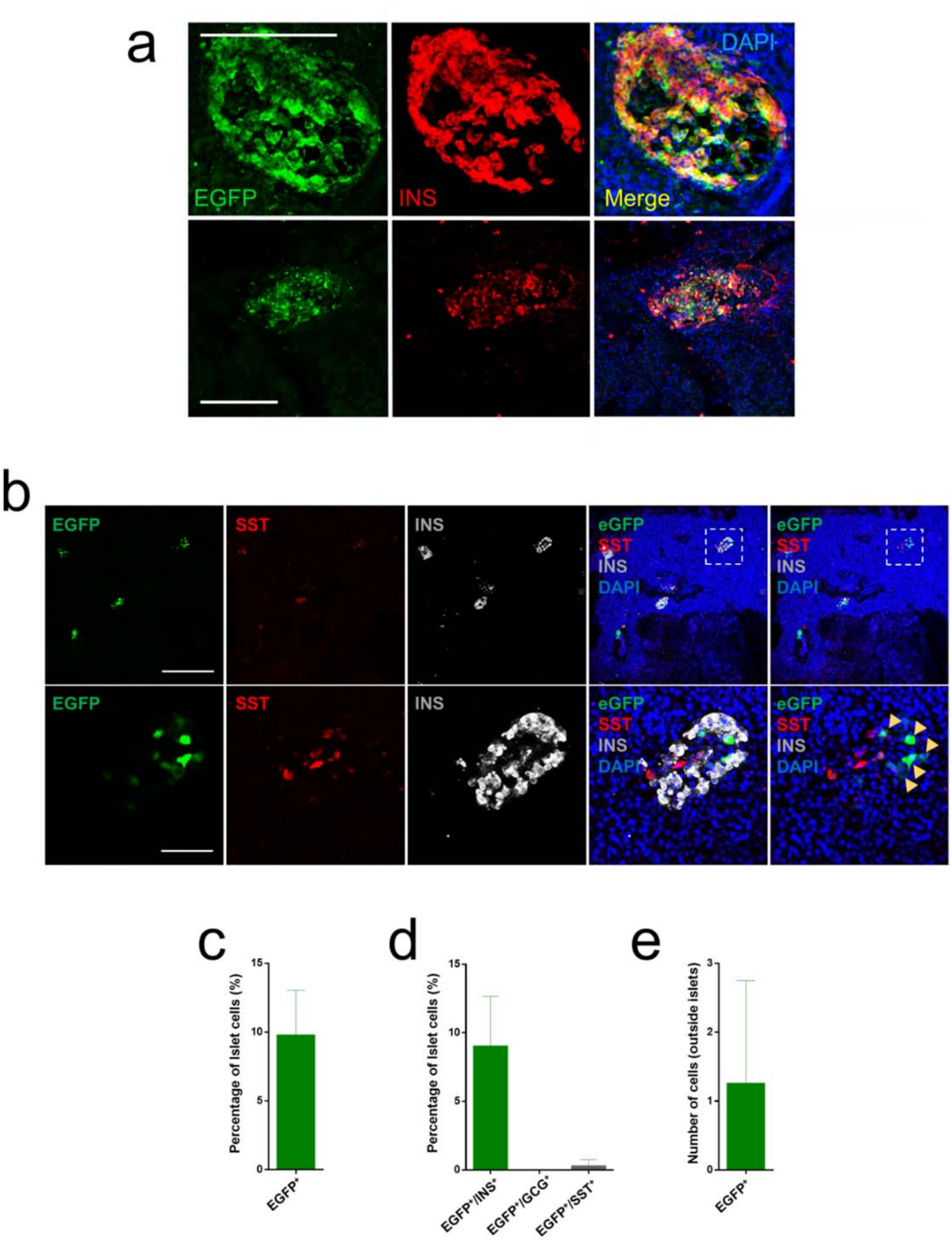

Supplementary Figure 5. Synthetic human Insulin promoter (SHIP) is β-cell specific in human pancreatic slices. (a) Top and bottom row: representative confocal immunofluorescence images at two different magnifications of a human pancreatic slice transduced with SHIP-EGFP and counterstained after 48 hours with insulin (n = 4). **(b)** Representative confocal immunofluorescence imaging of a region of a human pancreatic

slice containing islet-resident β-cells tagged by a SHIP-EGFP adenovirus. The lower row shows a magnified region for each channel of an islet with untagged δ-cells (SST, somatostatin$^+$), whereas some β-cells (insulin$^+$) are labeled by SHIP-EGFP (n = 4). **(c)** Percentage of EGFP-expressing islet cells (n = 4). **(d)** Percentage of islet cells expressing EGFP and either insulin, glucagon or somatostatin (n = 4) **(e)** Number of cells outside islet structures also expressing EGFP (n = 4). Size bar: 100 µm **(a)** and 200 µm **(b**, top row**)**; magnified (lower) panels in **(b)**: 50 µm. Data plotted in (**c-e**) are presented as mean ± SD. Plotted bars are centered at mean.

Supplementary Table 1. Pancreatic donor demographics

Sample ID	Diabetes Duration (Years)	Auto Antibody Assay (RIA)	C-Peptide (ng/ml)	Age (Years)	Sex	Ethnicity	BMI
HP2298	0	N/A	N/A	49	M	Hispanic	25.9
HP2305	5/T2D	N/A	N/A	59	M	Hispanic	30.7
HP2306	0	N/A	N/A	50	F	Caucasian	24.7
HP2307	5/T2D	N/A	N/A	54	F	Caucasian	28.1
HP2309	0	N/A	N/A	57	F	African American	29.2
HP2311	0	N/A	N/A	59	F	African American	21.7
HP2311	0	N/A	N/A	44	F	Caucasian	29.5
HP2315	(T2D)	N/A	N/A	61	M	Hispanic	33.7
HP2316	0	N/A	N/A	60	M	Caucasian	34.8
nPOD6461	0	Neg	5.26	14	M	Caucasian	18.5
nPOD6462	0	Neg	7.22	11.09	F	Caucasian	15.2
nPOD6465	0	Neg	3.42	4	M	African American	16.8
nPOD6468	0	Neg	5.27	16	M	Caucasian	15.9
nPOD6469	1.5 (T1D)	GADA A+	0.66	27	F	Caucasian	26.9
HP19-01	0	N/A	N/A	69	M	N/A	22.7
HP19-02	0	N/A	4.9	22	M	Caucasian	20.9
HP19-03	0	N/A	N/a	49	M	Caucasian	26.1
nPOD6516	0	N/A	5.5	20	M	Caucasian	28.8
HP19-05	0	N/A	5.3	64	M	Caucasian	17.6
HP20-01	0	N/A	4.7	28	M	Hispanic	20.3
HP20-02	0	N/A	5.1	31	M	N/A	31.9
HP20-03	0	N/A	4.9	42	M	Caucasian	28.1
HP20-04	0	N/A	N/A	54	M	N/A	29.4

Supplementary Table 2. Table of antibodies used.

Antibody to	Concentration	Host	Company	Catalogue number	Reactivity to human
	IF				
Insulin	1:250	Guinea Pig	Dako/Agilent	A0564	+++
Glucagon	1:500	Mouse	R&D Systems	MAB1249	+++
Glucagon	1:250	Rabbit	Dako/Agilent	A0565	+++
Cytokeratin19	1:100	Rabbit	Abcam	ab52625	+++
Cytokeratin19	1:100	Mouse	Dako	M0888	+++
Somatostatin	1:50	Rat	Millipore	MAB354	+++
Somatostatin	1:250	Rabbit	Dako/Agilent	A0566,	+++
Alpha Amylase	1:100	Rabbit	Sigma Aldrich	A8273	+++
Fluo-4, AM, cell permeant	10uM	Calcium Indicator	Thermo Scientific	F14201	+++
Ki-67 Antibody	1:50	Rabbit	Sigma Aldrich	AB9260	+++
E-Cadherin	1:250	Mouse	R&D Systems	AF748	+++
NKX6.1	1:100	Mouse	R&D Systems	AF5857	+++
PDX1	15 µg/ml	Goat	R&D Systems	AF2419	+++
