# Supplementary material for: Long-term culture of human pancreatic slices as a model to study real-time islet regeneration
Source: Nat Commun. 2020 Jun 29;11:3265. doi: 10.1038/s41467-020-17040-8 (PMC7324563; doi:10.1038/s41467-020-17040-8)
Supplement: Supplementary file 3 — Description of Additional Supplementary Files [file 41467_2020_17040_MOESM3_ESM.pdf]

Description of Additional Supplementary Files

Title: Supplementary Data 1

Description: Table of key resources used for this research.

Title: Supplementary Movie 1

Description: Dynamic modeling of oxygen diffusion through HPSs cultured atop PFC membranes.

Title: Supplementary Movie 2

Description: Dynamic modeling of oxygen diffusion through HPSs cultured atop transwells.

Title: Supplementary Movie 3

Description: Imaging of the Ca2+ stimulation of an islet within a human pancreatic slice cultured for 10 days atop a PFC membrane. The video shows stimulation with 16.7 mM glucose and subsequently KCl depolarization.

Title: Supplementary Movie 4

Description: Imaging of the Ca2+ stimulation of a human pancreatic slice cultured for 10 days atop a PFC membrane with KCl and carbachol. Only the islet featured in the field responds to the former, whereas only the surrounding exocrine tissue responds to the latter.
