# Supplementary material for: Long-term culture of human pancreatic slices as a model to study real-time islet regeneration
Source: Nat Commun. 2020 Jun 29;11:3265. doi: 10.1038/s41467-020-17040-8 (PMC7324563; doi:10.1038/s41467-020-17040-8)
Supplement: Supplementary file 4 — Supplementary Data 1 [file 41467_2020_17040_MOESM4_ESM.docx]

**SUPPLEMENTARY DATA 1. Table of key resources used for this research.**

| **REAGENT or RESOURCE** | **SOURCE** | **IDENTIFIER** |
| --- | --- | --- |
| **Antibodies** | | |
| Anti-BMPR1A-AF647-Rabbit Polyclonal | Bioss | Cat# bs-1509R-A647;  RRID: AB_2801504 |
| Anti-BMPR1A-Mouse Polyclonal | LS Biosciences | Cat# LS-C191759;  RRID: AB_2801506 |
| Anti-BMPR1A-Rabbit Polyclonal | Abcam | Cat# ab38560;  RRID: AB_722713 |
| Anti-P2RY1-Rabbit Polyclonal | LS Biosciences | Cat# LS-A387;  RRID: AB_592423 |
| Anti-P2RY1-FITC-Rabbit Polyclonal | Biorbyt | Cat# orb16131;  RRID: AB_1073728 |
| Anti-PDX1-Goat Polyclonal | R&D Systems | Cat# AF2419;  RRID: AB_355257 |
| Anti-NKX6.1-Mouse Polyclonal | R&D Systems | Cat# AF5857.  RRID: AB_1857045 |
| Anti-INSULIN-Guinea Pig Polyclonal | Dako/ Agilent | Cat# A0564;  RRID: AB_10013624 |
| Anti-INSULIN-Guinea Pig Polyclonal, FLEX Ready to use | Dako/ Agilent | Cat# IR002  RRID: AB_2800361 |
| Anti-Glucagon-Mouse Polyclonal | R&D Systems | Cat# MAB1249;  RRID: AB_2107340 |
| Anti-Glucagon-Rabbit Polyclonal | Dako/ Agilent | Cat# A0565;  RRID: AB_10013726 |
| Anti- E-Cadherin- Mouse Polyclonal | R&D Systems | Cat# AF748,  RRID: AB_355568 |
| Anti-KRT19-Rabbit Monoclonal | Abcam | Cat# ab52625;  RRID: AB_2281020 |
| Anti-KRT19-Mouse Polyclonal | Dako/ Agilent | Cat# M0888;  RRID: AB_2234418 |
| Anti-Somatostatin-Rat Polyclonal | Millipore | Cat# MAB354  RRID: AB_2255365 |
| Polyclonal Rabbit Anti-Human Somatostatin antibody | Dako/ Agilent | Cat# A0566,  RRID: AB_2688022 |
| Anti-Alpha Amylase-Rabbit Polyclonal | Sigma Aldrich | Cat# A8273;  RRID: AB_258380 |

| Anti-Ki-67 Antibody- Rabbit Polyclonal | Millipore/Sigma | Cat# AB9260  RRID: AB_2142366 |
| --- | --- | --- |
| Alexa Fluor® 647 AffiniPure Donkey Anti-Guinea Pig IgG (H+L) | Jackson Immuno Research laboratories, Inc. | Cat# 706-605-148;  RRID: AB_2340476 |
| Alexa Fluor Donkey polyclonal anti-rabbit IgG, Alexa Fluor 488 | Thermofisher Scientific | Cat# A-21206;  RRID: AB_2535792 |
| Donkey Anti-Sheep IgG (H+L) Antibody, Alexa Fluor 488 Conjugated | Thermofisher Scientific | Cat# A-11015,  RRID: AB_141362 |
| Donkey Anti-Rabbit IgG (H+L) Polyclonal Antibody, Alexa Fluor 647 Conjugated | Thermofisher Scientific | Cat# A-31573,  RRID: AB_2536183 |
| Donkey anti-Mouse IgG (H+L) Highly Cross-Adsorbed Secondary Antibody, Alexa Fluor 594 | Thermofisher Scientific | Cat# A-21203,  RRID: AB_2535789 |
| Donkey anti-Goat IgG (H+L) Cross-Adsorbed Secondary Antibody, Alexa Fluor 647 | Thermofisher Scientific | Cat# A-21447,  RRID: AB_2535864 |
| Donkey anti-Rabbit IgG (H+L) Highly Cross-Adsorbed Secondary Antibody, Alexa Fluor 594 | Thermofisher Scientific | Cat# A-21207,  RRID: AB_141637 |
| Donkey anti-Goat IgG (H+L) Cross-Adsorbed Secondary Antibody, Alexa Fluor 488 | Thermofisher Scientific | Cat# A-11055  RRID: AB_2534102 |
| Donkey anti-Mouse IgG (H+L) Highly Cross-Adsorbed Secondary Antibody, Alexa Fluor 647 | Thermofisher Scientific | Cat# A-31571  RRID: AB_162542 |

| **Biological Samples** | | |
| --- | --- | --- |
| Human Pancreas Exocrine Tissue | cGMP Facility, Diabetes Research Institute, Miami, FL | http://www.diabetesresearch.org/cGMP-GTP-cell-processing |
| Human Pancreas Slices | nPOD, University of Florida, Gainesville, FL | [https://www.jdrfnpod.org](about:blank) |
| Human Pancreas Tissue | Prodo Laboratories INC,  Aliso Viejo, CA | <https://prodolabs.com/> |
| Human Pancreas Tissue | University of Alberta IsletCore, Edmonton, Canada | <https://www.ualberta.ca/alberta-diabetes/core-services/isletcore> |
| **Chemicals, Peptides, and Recombinant Proteins** | | |
| Trypsin inhibitor isolated from *Glycine max* | Sigma Aldrich | Cat# T6522 |
| DPBS | Sigma Aldrich | Cat# D8537 |
| Fetal bovine serum | Thermofisher Scientific | Cat# 10082147 |
| RPMI 1640 containing GlutaMAX | Thermofisher Scientific | Cat# 61870036 |
| HEPES buffer | Thermofisher Scientific | Cat# 15630080 |
| Aprotinin | Sigma Aldrich | Cat# A6106;  CAS# 9087-70-1 |
| Penicillin-streptomycin-amphotericin B solution | Sigma Aldrich | Cat# A5955 |
| Chymostatin | Sigma Aldrich | Cat# 11004638001 |
| Carbachol | Sigma Aldrich | Cat# 212385-M |
| Cholecystokinin 8 | Sigma Aldrich | Cat# C2901 |
| Sodium Pyruvate | Thermofisher Scientific | Cat# 11360070 |
| D-glucose | Sigma Aldrich | Cat# G8644 |
| B27-minus insulin | Thermofisher Scientific | Cat# A1895601 |
| Fibronectin | Sigma Aldrich | Cat# F1141 |
| Collagen type-I | Sigma Aldrich | Cat# A10483-01 |
| Waymouth’s MB 752/1 medium | Biological Industries | Cat# 06-1110-01-1A |
| Basal BrainPhys neuronal medium | Stemcell Technologies | Cat# 05790 |
| L-Glutamic Acid | Sigma Aldrich | Cat# 49449 |
| Glutamax supplement | Invitrogen | Cat# 35050061 |
| Tryple-E Express Enzyme | Thermofisher Scientific | Cat# 12604013 |
| Corning® Matrigel® hESC-Qualified Matrix, *LDEV-free | Corning | Cat# 354277 |
| DAPI (4',6-Diamidino-2-Phenylindole, Dihydrochloride) | Thermofisher Scientific | Cat# 1306 |
| Fluo-4 AM | Invitrogen | Cat# F14201 |
| ProLong™ Gold Antifade Mountant | Thermofisher Scientific | Cat#: P36930  RRID:AB_2801517 |
| Nuclear Decloaker, 10X | BioCare Medical | Cat # CB911M  RRID: AB_2801515. |
| Antigen Decloaker, 10X | BioCare Medical | Cat # CB910M  RRID: AB_2801528 |
| Power block | Biogenex | Cat# HK085-5K |
| Protein Block, Serum-Free | Dako/ Agilent | Cat # X090930-2  RRID: AB_2801516. |
| ImmEdge Pen | Vector Laboratories | Cat# H-4000,  RRID: AB_2336517 |
| Normal Donkey Serum antibody | Jackson Immuno Research laboratories, Inc. | Cat# 017-000-121  RRID: AB_2337258 |
| Out the Door-Top Coat | International Nail Manufacturers, (inm) | Cat#S401025  RRID: AB_2801526. |
|  |  |  |
| **Critical Commercial Assays** | | |
| LIVE/DEAD™ Fixable Near-IR Dead Cell Stain Kit, for 633 or 635 nm excitation | Invitrogen | Cat# L34975 |
| Contour Next Ez Blood Glucose Monitoring Kit | Bayer | Cat# 9628;  UPC# 301939628014 |
| Live/Dead viability/cytotoxicity kit for mammalian cells | Invitrogen | Cat# L3224 |
| Quant-iT Picogreen dsDNA assay kit | Invitrogen | CAT# P7589 |
| Mercodia Ultrasensitive C-peptide ELISA | Mercodia | Cat# 10-1141-01 |
| Mercodia Human Insulin ELISA | Mercodia | Cat# 10-1113-10 |
| Colorimetric assay for amylase activity | BioVision | Cat# K711-100 |

| Glucagon ELISA KIT | Crystal Chem | Cat# 81520 |
| --- | --- | --- |
| Human Hypoxia-inducible factor 1-alpha ELISA Kit | MyBioSource | Cat# MBS2885065 |
| Human/Mouse Total HIF-1 alpha/HIF1 alpha DuoSet IC | R&D Systems | Cat# DYC1935-2 |
| Pierce™ BCA Protein Assay Kit | Thermo Fisher | Cat# 23227 |
| Custom TaqMan Array Cards – Human pancreatic array | Thermo Fisher | Cat# 4342253 |

| **Deposited Data** | | |
| --- | --- | --- |
| N/A |  |  |
| **Experimental Models: Organisms/Strains** | | |
| B6.Cg-Tg(Ins2-cre)25Mgn/J | The Jackson Laboratory | Cat# 003573;  MGI: J:51826;  https://www.jax.org/strain/003573 |
| B6.129(Cg)-Gt(ROSA)26Sortm4(ACTB-tdT,-eGFP)Luo | The Jackson Laboratory | Cat# 007676;  MGI: J:124702;  https://www.jax.org/strain/007676 |
| B6.Cg-Tg(Ins1-EGFP)1Hara/J | The Jackson Laboratory | Cat# 006864;  MGI: J:99450;  https://www.jax.org/strain/006864 |
| CD1-IGS | Charles River | Cat# 022  <https://www.criver.com/products-services/find-model/cd-1r-igs-mouse?region=3611> |
|  |  |  |
| **Software and Algorithms** | | |
| Fiji ImageJ | (Schindelin et al., 2012) | <https://fiji.sc/> |
| GraphPad Prism v8 | GraphPad | <https://www.graphpad.com/scientific-software/prism/> |
| Summit software v6.3 | Beckman Coulter | <https://www.beckman.com/flow-cytometry> |
| Kaluza v1.5a and v2.1.1 | Beckman Coulter | <https://www.beckman.com/flow-cytometry/software/kaluza> |
| CentOS 6.5 (64x bit, for Windows) | linux | <http://vault.centos.org/6.5/isos/x86_64/> |
| Windows 10 Professional (64x bit) | Microsoft | <https://www.microsoft.com/en-us/p/windows-10-pro/df77x4d43rkt?activetab=pivot%3aoverviewtab> |
| AxioVision v4.6 (32x bit, for Windows) | Zeiss | <https://www.micro-shop.zeiss.com/en/us/system/software+axio+vision-axiovision+program-axiovision+software/10221/> |
| COMSOL v5.3 | COMSOL Inc. | <https://www.comsol.com/product-download> |
| Leica Application Suite (LAS) v5 | Leica Microsystems | <https://www.leica-microsystems.com/products/microscope-software/p/leica-application-suite/> |
| **qRT-PCR primers** |  | Assay # & context sequence |
| B2M | Applied Biosystems/Thermo Fisher Scientific | Hs99999907_m1  GTTAAGTGGGATCGAGACATGTAAG |
| CDH1 | Applied Biosystems/Thermo Fisher Scientific | Hs01013953_m1  GAACAGAAAATAACATATCGGATTT |
| EPCAM | Applied Biosystems/Thermo Fisher Scientific | Hs00901885_m1  AGTGAGAACCTACTGGATCATCATT |
| PNLIP | Applied Biosystems/Thermo Fisher Scientific | Hs00609591_m1  TCTTCAGTCGGCGTTCGGTTACTCA |
| CPA1 | Applied Biosystems/Thermo Fisher Scientific | Hs00156992_m1  ACTTTGTGGGGCATCAGGTGCTCCG |
| INS | Applied Biosystems/Thermo Fisher Scientific | Hs00355773_m1  CTGCAGGTGGGGCAGGTGGAGCTGG |
| GCG | Applied Biosystems/Thermo Fisher Scientific | Hs01031536_m1  CCAAGAGGAACAGGAATAACATTGC |
| SST | Applied Biosystems/Thermo Fisher Scientific | Hs00174949_m1  GCGGGGAAGCAGGAACTGGCCAAGT |
| PPY | Applied Biosystems/Thermo Fisher Scientific | Hs00237001_m1  TGCTGTCCCCAGGGAGCTCAGCCCG |
| IAPP | Applied Biosystems/Thermo Fisher Scientific | Hs00169095_m1  TTGAAAGTCATCAGGTGGAAAAGCG |
| MAFA | Applied Biosystems/Thermo Fisher Scientific | Hs01651425_s1  CGCTCATCGGCAGCGGCCACCACGG |
| NEUROD1 | Applied Biosystems/Thermo Fisher Scientific | Hs00159598_m1  ACAAAGGAAATCGAAACATGACCAA |
| NKX6.1 | Applied Biosystems/Thermo Fisher Scientific | Hs00232355_m1  CCTGTACCCCTCATCAAGGATCCAT |
| PDX1 | Applied Biosystems/Thermo Fisher Scientific | Hs00236830_m1  CAGTGGGCAGGCGGCGCCTACGCTG |
| SLC2A1 | Applied Biosystems/Thermo Fisher Scientific | Hs01096908_m1  CACTGCTGTCTCTGTATTCCTTGTG |
| CHGA | Applied Biosystems/Thermo Fisher Scientific | Hs00900370_m1  GGGGATACCGAGGTGATGAAATGCA |
| MUC6 | Applied Biosystems/Thermo Fisher Scientific | Hs01674026_g1  AAGCCCCACGCTGCCAAAATCGACC |
| **Other** | | |
| Donor Demographics | This manuscript | Supplementary Table 1 |
| Table of antibodies used | This manuscript | Supplementary Table 2 |

**SUPPLEMENTAL REFERENCES**

1. Schindelin, J., Arganda-Carreras, I., Frise, E., Kaynig, V., Longair, M., Pietzsch, T., Preibisch, S., Rueden, C., Saalfeld, S., and Schmid, B. (2012). Fiji: an open-source platform for biological-image analysis. Nature Methods *9*, 676.
