# Supplementary figures and images for: Long-term culture of human pancreatic slices as a model to study real-time islet regeneration
Source: Nat Commun. 2020 Jun 29;11:3265. doi: 10.1038/s41467-020-17040-8 (PMC7324563; doi:10.1038/s41467-020-17040-8)

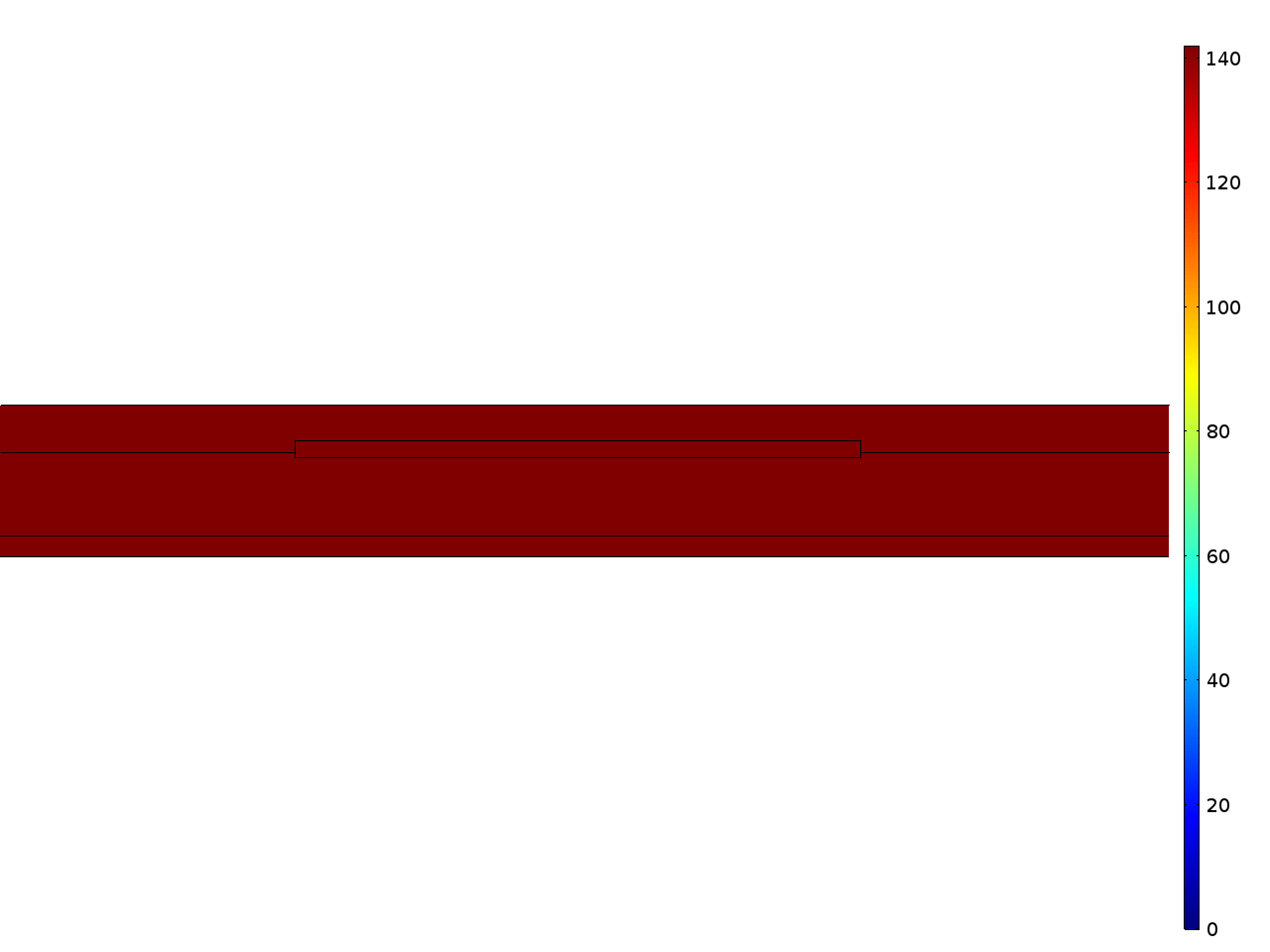

Supplement: Supplementary file 5 — Supplementary Movie 1 [file 41467_2020_17040_MOESM5_ESM.gif]

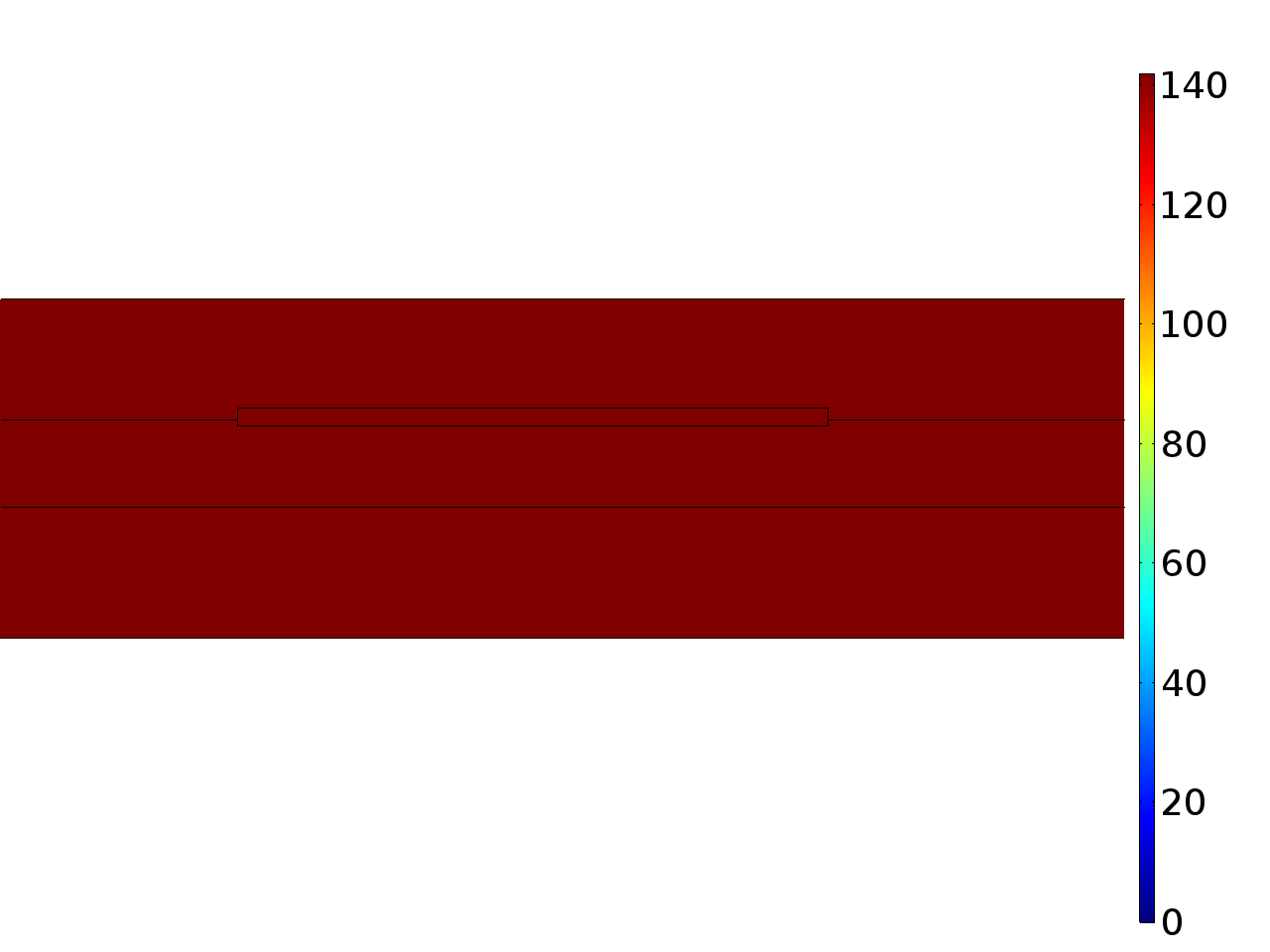

Supplement: Supplementary file 6 — Supplementary Movie 2 [file 41467_2020_17040_MOESM6_ESM.gif]
