# Supplementary material for: Long-term culture of human pancreatic slices as a model to study real-time islet regeneration
Source: Nat Commun. 2020 Jun 29;11:3265. doi: 10.1038/s41467-020-17040-8 (PMC7324563; doi:10.1038/s41467-020-17040-8)
Supplement: Supplementary file 9 — Reporting Summary [file 41467_2020_17040_MOESM9_ESM.pdf]

nature research

Corresponding author(s): JUAN DOMINGUEZ-BENDALA

Last updated by author(s): MAY 27, 2020

Reporting Summary

Nature Research wishes to improve the reproducibility of the work that we publish. This form provides structure for consistency and transparency in reporting. For further information on Nature Research policies, see our Editorial Policies and the Editorial Policy Checklist.

Statistics

For all statistical analyses, confirm that the following items are present in the figure legend, table legend, main text, or Methods section.

n/a	Confirmed	
☐	☒	The exact sample size (n) for each experimental group/condition, given as a discrete number and unit of measurement
☐	☒	A statement on whether measurements were taken from distinct samples or whether the same sample was measured repeatedly
☐	☒	The statistical test(s) used AND whether they are one- or two-sided<br>*Only common tests should be described solely by name; describe more complex techniques in the Methods section.*
☒	☐	A description of all covariates tested
☐	☒	A description of any assumptions or corrections, such as tests of normality and adjustment for multiple comparisons
☐	☒	A full description of the statistical parameters including central tendency (e.g. means) or other basic estimates (e.g. regression coefficient) AND variation (e.g. standard deviation) or associated estimates of uncertainty (e.g. confidence intervals)
☐	☒	For null hypothesis testing, the test statistic (e.g. F, t, r) with confidence intervals, effect sizes, degrees of freedom and P value noted<br>*Give P values as exact values whenever suitable.*
☒	☐	For Bayesian analysis, information on the choice of priors and Markov chain Monte Carlo settings
☒	☐	For hierarchical and complex designs, identification of the appropriate level for tests and full reporting of outcomes
☒	☐	Estimates of effect sizes (e.g. Cohen's d, Pearson's r), indicating how they were calculated

Our web collection on statistics for biologists contains articles on many of the points above.

Software and code

Policy information about availability of computer code

Data collection	All data in this study was collected via commercial software as described in the appropriate methods section. Media analytes were measured using a Beckman Coulter Vi-Cell MetaFLEX. Longitudinal imaging was performed using a ApoTome Axiovert 200M (Zeiss) fluorescent microscope. Endpoint imaging was performed using a Leica MP-NDD4/SP5/FCS/FLIM multiphoton/confocal upright F-techniques microscope MP/SP5 with a laser set at 30% intensity, pinhole set at 1, airy set at 1, with 10X, 20X, 40X and 63X magnification settings; and dynamic Calcium responses were recorded using a Leica SP5 multi-photon confocal laser-scanning microscope with 40X magnification (NA = 0.8). Dynamic perifusion assays were performed using a Biorep PERI3 perifusion machine. ELISAs, BCA assays and ds-gDNA quantifications were perfomed using commercially available kits and read using a Biotek plate reader. qRT-PCRs were performed utilizing Applied biosystems kits and the 7900HT and StepOnePlus real time PCR systems.
Data analysis	Data acquisition for imaging was performed using the Leica Application Suite v5 (Leica, Wetzlar, Germany); Fiji Image J v1.52p (NIH, USA); AxioVision v4.6 and Carl Zeiss Zen Pro v2.6 softwares (Carl Zeiss Microscopy, Oberkochen, Germany). Metabolic Modeling was performed using COMSOL v5.3 (Comsol Inc, Burlington, MA). Data Analysis was performed using Graphpad Prism v6 and v8 (Graphpad Software Inc.) and Microsoft Excel (Microsoft, USA). Source data for figures 1-5 is provided in the source data file.

For manuscripts utilizing custom algorithms or software that are central to the research but not yet described in published literature, software must be made available to editors and reviewers. We strongly encourage code deposition in a community repository (e.g. GitHub). See the Nature Research guidelines for submitting code & software for further information.

Data

Policy information about availability of data

All manuscripts must include a data availability statement. This statement should provide the following information, where applicable:

- Accession codes, unique identifiers, or web links for publicly available datasets
- A list of figures that have associated raw data
- A description of any restrictions on data availability

Raw data for Figures 1-5 have been made available in the source datafile.

Field-specific reporting

Please select the one below that is the best fit for your research. If you are not sure, read the appropriate sections before making your selection.

☒ Life sciences ☐ Behavioural & social sciences ☐ Ecological, evolutionary & environmental sciences

For a reference copy of the document with all sections, see nature.com/documents/nr-reporting-summary-flat.pdf

Life sciences study design

All studies must disclose on these points even when the disclosure is negative.

Sample size	No calculations for sample size were performed. Owing to limited human pancreatic slices, we optimized their number per experiments (n>3 donors/experiment and n≥3 slices per donor), unless using precious tissue from diabetic donors, where we were only able to limit 3/slices per data point owing to a very limited number of slices available. For mouse studies we utilized n=5 (individual pancreas) and for each n at least 3 slices.
Data exclusions	Slices that were inefficiently transfected with adenovirus (low amount of red-fluorescence <5% of total slice area at day 5-10), or which showed non-specific tagging to adenovirus (eGFP signal >3% in mouse or >0.15% in human at day 5-10, confirmed via Insulin staining) were excluded and not analyzed. There are instances when human pancreatic slices do not get transfected efficiently with adenoviruses, possibly due to early necrosis and slice tissue death. The analysis of such slices results in analytical bias, so a strict range of fluorescence characteristics must be defined for any particular longitudinal regeneration experiment. We were unable to attain high transfection efficiencies with lentiviral or adeno-associated viral constructs.
Replication	All experiments were performed independently, by non-blinded investigators. Data was reproducible for the donor slices having high viability and appropriate transfection efficiency, at the start of the experiment. The experimental predictions were confirmed in every case, but a great donor-to-donor variability was observed, and has been reported in our analysis. Replication was unsuccessful when low transfection efficiency was observed. For GSIS data, experiments were completely repeated by an investigator independent of the corresponding author's lab and were reproduced.
Randomization	Allocation of slices to every experiment was completely randomized.
Blinding	Blinding not possible. Due to the nature of the pancreatic samples and the dissimilar culture conditions, the investigators were aware of the identity of the groups.

Reporting for specific materials, systems and methods

We require information from authors about some types of materials, experimental systems and methods used in many studies. Here, indicate whether each material, system or method listed is relevant to your study. If you are not sure if a list item applies to your research, read the appropriate section before selecting a response.

Materials & experimental systems

n/a	Involved in the study	
☐	☒	Antibodies
☒	☐	Eukaryotic cell lines
☒	☐	Palaeontology and archaeology
☐	☒	Animals and other organisms
☐	☒	Human research participants
☒	☐	Clinical data
☒	☐	Dual use research of concern

Methods

n/a	Involved in the study	
☒	☐	ChIP-seq
☒	☐	Flow cytometry
☒	☐	MRI-based neuroimaging

Antibodies

Antibodies used	anti-human/mouse Insulin (Dako/Agilent cat# A0564), anti-human/mouse Glucagon (R&D Systems cat# MAB1249), anti-human/

	mouse Glucagon (Dako/Agilent cat# A0565), anti-human/mouse Cytokeratin19 (Abcam cat# ab52625), anti-human/mouse Cytokeratin19 (Dako cat# M0888), anti-human/mouse Somatostatin (Millipore cat# MAB354), anti-human/mouse Somatostatin (Dako/Agilent cat# A0566), anti-human/mouse alpha-amylase (Sigma Aldrich cat# A8273), Fluo-4, AM cell permeant (Thermoscientific Cat# F14201).Ki-67 Antibody 1:50 Rabbit Sigma Aldrich AB9260; E-Cadherin 1:250 Mouse R&D Systems AF748; NKX6.1, 1:100 Mouse R&D Systems AF5857; PDX1 15 µg/ml Goat R&D Systems AF2419. Antibody dilutions used in this study are present in supplemental table 2.
Validation	All antibodies used in our studies, historically have been highly cited in the field and validated by multiple recognized laboratories and institutions. We have focused on citation lists as validation to support our claim.<br><br>Anti-human/mouse Insulin (Dako/Agilent cat# A0564) has over 350 citations (https://www.citeab.com/antibodies/3382917-a0564-insulin); anti-human/mouse Glucagon (R&D Systems cat# MAB1249) has 4 citations (https://www.rndsystems.com/products/human-mouse-glucagon-antibody-181402_mab1249), anti-human/mouse Glucagon (Dako/Agilent cat# A0565) has 8 citations (https://www.labome.com/product/Dako/A0565.html), anti-human/mouse Cytokeratin19 (Abcam cat# ab52625) has over 50 citations (https://www.abcam.com/cytokeratin-19-antibody-ep1580y-cytoskeleton-marker-ab52625.html), anti-human/mouse Cytokeratin19 (Dako cat# M0888) has over 50 citations (https://www.citeab.com/antibodies/2414887-m0888-cytokeratin-19-concentrate), anti-human/mouse Somatostatin (Millipore cat# MAB354) has over 200 citations (https://www.citeab.com/antibodies/226135-mab354-anti-somatostatin-antibody-clone-yc7), anti-human/mouse Somatostatin (Dako/Agilent cat# A0566) has over 50 citations (https://www.citeab.com/antibodies/3382932-a0566-somatostatin), anti-human/mouse alpha-amylase (Sigma Aldrich cat# A8273) has over 50 citations (https://www.sigmaaldrich.com/catalog/product/sigma/a8273?lang=en&region=US), Fluo-4, AM cell permeant (Thermoscientific Cat# F14201) has over 300 citations (https://www.thermofisher.com/search/results?query=F14201&persona=DocSupport&type=Citations+%26+References). Ki-67 Antibody 1:50 Rabbit (Sigma Aldrich AB9260) has over 100 citations; E-Cadherin 1:250 (Mouse R&D Systems AF748) has over 50 citations (https://www.citeab.com/antibodies/694006-af748-human-mouse-e-cadherin-antibody); NKX6.1, 1:100 (Mouse R&D Systems AF5857) has 8 citations (https://www.citeab.com/antibodies/692713-af5857-human-mouse-nkx6-1-antibody); PDX1 15 µg/ml Goat (R&D Systems AF2419) has over 10 citations (https://www.citeab.com/antibodies/2494241-af2419-pdx-1-ipf1-antibody-unconjugated).

Animals and other organisms

Policy information about studies involving animals; ARRIVE guidelines recommended for reporting animal research

Laboratory animals	All animal experiments were conducted under the supervision and oversight of the University of Miami Institutional Animal Care and Use Committee (IACUC) and Division of Veterinary Resources (DVR) at the University of Miami (https://www.uresearch.miami.edu/research-resources/dvr/index.html). Ambient temperature ranges between 75±4 °F and housed in specific pathogen-free (SPF) conditions at the DVR's animal care facility. For all experiments, mice were acclimated for 7-10 days prior to any experimental intervention. They were maintained on a 12h light/dark cycle with ad libitum access to standard irradiated chow and filtered acidified drinking water. B6.Cg-Tg(Ins2-cre)25Mgn/J (equal males and females, 10 weeks of age, all F1 generation mice from breeding stock), B6.129(Cg)-Gt(ROSA)26Sortm4(ACTB-tdT,-eGFP)Luo (equal males and females, 10 weeks of age, all F1 generation mice from breeding stock), B6.Cg-Tg(Ins1-EGFP)1Hara/J (equal males and females, 10 weeks of age, all F1 generation mice from breeding stock), CD1-IGS ((equal males and females, 10 weeks of age, all F1 generation mice from breeding stock), B6.Cg-Tg(Ins2-cre)25Mgn/J X Gt(ROSA)26Sortm4(ACTB-tdT,-eGFP)Luo (equal males and females, 10 weeks of age, all F1 generation mice from breeding stock)
Wild animals	No wild animals were used in the study.
Field-collected samples	No field collected samples were used in the study.
Ethics oversight	University of Miami, IACUC, DVR and IRB.

Note that full information on the approval of the study protocol must also be provided in the manuscript.

Human research participants

Policy information about studies involving human research participants

Population characteristics	N/A
Recruitment	N/A
Ethics oversight	N/A

Note that full information on the approval of the study protocol must also be provided in the manuscript.
